# A Novel Crow Swarm Optimization Algorithm (CSO) Coupling Particle Swarm Optimization (PSO) and Crow Search Algorithm (CSA)

**DOI:** 10.1155/2021/6686826

**Published:** 2021-05-22

**Authors:** Ying-Hui Jia, Jun Qiu, Zhuang-Zhuang Ma, Fang-Fang Li

**Affiliations:** ^1^College of Water Resources & Civil Engineering, China Agricultural University, Beijing 100083, China; ^2^State Key Laboratory of Hydroscience & Engineering, Tsinghua University, Beijing 100084, China; ^3^State Key Laboratory of Plateau Ecology and Agriculture, Qinghai University, Xining 810016, China

## Abstract

The balance between exploitation and exploration essentially determines the performance of a population-based optimization algorithm, which is also a big challenge in algorithm design. Particle swarm optimization (PSO) has strong ability in exploitation, but is relatively weak in exploration, while crow search algorithm (CSA) is characterized by simplicity and more randomness. This study proposes a new crow swarm optimization algorithm coupling PSO and CSA, which provides the individuals the possibility of exploring the unknown regions under the guidance of another random individual. The proposed CSO algorithm is tested on several benchmark functions, including both unimodal and multimodal problems with different variable dimensions. The performance of the proposed CSO is evaluated by the optimization efficiency, the global search ability, and the robustness to parameter settings, all of which are improved to a great extent compared with either PSO and CSA, as the proposed CSO combines the advantages of PSO in exploitation and that of CSA in exploration, especially for complex high-dimensional problems.

## 1. Introduction

Bio-inspired optimization algorithms have become increasingly popular over the past decade due to their simplicity of implementation, robustness, and ability of parallel computation [1]. Although the specific principles and procedures of bio-inspired optimization algorithms are various, it is a consensus that an effective search technique must strike a balance between exploring new regions in the search space and exploiting known promising regions [2, 3].

Particle swarm optimization (PSO), developed by Kennedy [4] in 1995, is one of the most popular bio-inspired algorithms with wide applications in industrial design [5], energy distribution [6], economic dispatch [7], and so on. PSO is inspired by the social behavior of bird flocking or fish schooling. A swarm of particles moves inside a bounded search space and cooperates to identify the best solution under the guidance of social attraction and cognitive attraction, which are used with the aim of exploiting and controlling the cooperation within the swarm. Meanwhile, the exploration of the search space is considered by an inertia factor weighing the movement of particles and the magnitude of their velocities. Nevertheless, similar to other bio-inspired algorithms, PSO also suffers from the bane of premature convergence and entrapment in local optima when solving complex multimodal problems [8, 9], i.e., the algorithm has difficulty exploring all regions, and some of the peaks are easily missed. Various improvements to PSO have been made to balance exploration and exploitation search.

Crow search algorithm (CSA), proposed by Askarzadeh [10] in 2016, is a newly developed algorithm inspired by the strategic behavior of crows while searching food, thievery, and chasing behavior. CSA is characterized by easy implementation, less parameter setting, and relatively strong development capacity in the searching process [11]. However, CSA also suffers from low search precision, high possibility of getting into the local optimum, and premature convergence, especially for multidimensional optimization problems [11], which may result from two important features in the basic CSA [12, 13]: (1) there is no criterion for choosing the destination and the selection is done randomly between all crows; (2) the amount of flight length is a constant value which may cause inappropriate searching by the crows in the solution space that results in trapping in the local optimum. In the past few years, most of the recent research studies focused on the application of the algorithm in different scientific fields with suitable modification. In case of feature selection, Sayed et al. [3] combined chaos with CSA to enhance the performance and convergence speed, while Chaudhuri and Sahu [14] proposed time varying flight length to obtain better results. As for energy optimization, Makhdoom and Askarzadeh [15] incorporated adaptive chaotic awareness probability into CSA to optimize operation of photovoltaic/diesel hybrid energy system. To overcome unbalanced exploration and exploitation phases, Shekhawat and Saxena [13] improved the algorithm by a cosine function and opposition-based learning concept. CSA variants have been categorized into modified versions [16–18] and hybrid versions [19–21].

Both PSO and CSA are population-based techniques. For optimization problems, the most critical part is to find as many local optimal solutions as possible and gradually move to the global best. Therefore, the most important thing is to give the solutions some degree of freedom during iteration while making the optimization process efficient, i.e., to balance between exploration and exploitation. It can be inferred from the previous studies that PSO is more capable in controlling exploitation by social and cognitive factors, but its ability of exploration in the unknown region is highly influenced by the current best solutions in spite of the existence of random numbers affecting the acceleration coefficients. In contrast, the individual in CSA moves completely randomly when it is aware of being tracked to get rid of the stalker. Hence, CSA performs better in exploring new regions in the search space. A few studies have proposed hybrid models combining PSO and CSA. Babu et al. [22] have proposed a model that connects the update procedure of CSA with that of PSO, i.e., every particle updates its position twice for each iteration. Crow Search Algorithm Auto-Drive PSO [23] uses CSA as the outer algorithm to optimize the sizing of renewable distributed generations; then, PSO is applied for the optimal power flow of the power distribution system as inner optimization. Both algorithms execute the position updating procedure using formulas from PSO and CSA during each iteration. In [22], it is used to address the same problem, while Farh et al. [23] divide a problem into two parts which are optimized by CSA and PSO, respectively. However, their algorithms are applied on specific situations without detailed evaluation on further performance. To take advantage of the ability of CSA in exploration and the ability of PSO in exploitation, this paper proposed a new Crow Swarm Optimization algorithm (CSO). Besides, moving towards the (current) best particle in the swarm and the best position is autonomously found so far, and each individual also has a probability to stalk the best ever solution of another individual. The proposed CSO essentially adds the information sharing mechanism in CSA and explores the unknown region between the two individuals using PSO. Numerical benchmark problems are tested using the proposed algorithm, and the results prove that it performs better in balancing exploitation and exploration with a few parameters.

The rest of the paper is organized as follows.  Section 2 introduces the proposed CSO algorithm as well as traditional PSO and CSA.  Section 3 is the performance evaluation on several benchmark functions with an example of application in real-world problem.  Section 4 draws the conclusions.

## 2. Crow Swarm Optimization Algorithm (CSO)

### 2.1. Standard PSO

PSO imitates the foraging behavior of bird flocks and consists of a collection of agents, so-called particles. The position of each particle represents a solution, and each particle retains the information of its current position, velocity, and its personal best found position within the search space. The particles move to new positions with a velocity vector, which is iteratively updated based on its attraction towards the best position found by the particle and the best position found by any particle within the search space, i.e., the position of particle *i* at the *t*th iteration is determined by the position vector *x*_*i*_^*t*^ and the velocity vector *v*_*i*_^*t*^. The updating formulas for particle velocity and position are shown in Equations (1) and (2):

(1) $$\begin{array}{c} v_{i}^{t+1}=\omega v_{i}^{t}+c_{1}r_{1}\left(pb_{i}^{t}-x_{i}^{t}\right)+c_{2}r_{2}\left(gb^{t}-x_{i}^{t}\right),\;i=1,2,\ldots ,n\;v_{\min}\leq v_{i}^{t+1}\leq v_{\max}, \end{array}$$

(2) $$\begin{array}{c} x_{i}^{t+1}=x_{i}^{t}+v_{i}^{t+1},x_{\min}\leq x_{i}^{t+1}\leq x_{\max}, \end{array}$$

where *n* is the number of the particles in population, *ω* is the inertia weight coefficient, *c*_1_ and *c*_2_ are called acceleration coefficients, indicating the cognition degree of the particle to the individual and the society, respectively,  *r*_1_ and *r*_2_ are random numbers in [0, 1], **pb**_*i*_^*t*^ represents the current optimal position of *i* particle, **gb**^*t*^ represents the best position of the current population, **v**_min_ and **v**_max_ are the lower and upper limit of particle update velocity, respectively; in this paper, **v**_min_=−**v**_max_. **x**_min_ and **x**_max_ are the minimum and maximum positions of particles, respectively.

The key idea of PSO is that, under the dominant action of better particles, each particle is close to the global optimal position. Although there exist random numbers *r*_1_ and *r*_2_ to avoid that the movements of the particles are completely decided by its current optimal position and the best position of the current population, it can be inferred that these two factors have great impact on the movement of particles. Thus, the initial position of the particles affects the search process greatly, and a large part of unknown regions can be hardly paid attention to. Such characteristic provides a strong capacity of exploiting the known information, while it is weaker in the exploration of new regions, which lead to premature convergence, i.e., the optimization easily converges to a local optimum.

### 2.2. Standard CSA

CSA is a new search algorithm inspired by the behavior of crows hiding and stealing food. It is nongreedy and can increase the variety of generated solutions. The principles of CSA are listed as follows: (1) crows live in the form of flock, (2) each crow can memorize the position of their hiding places and steal food from other crows, and (3) with a certain probability, the crow can be aware of being stalked and then protect its food from being stolen by flying randomly. The position of each crow represents a solution **x**_*i*_^*t*^. During the iteration, crow *i*  tracks a random crow  *j*. If crow *j* is not aware of being tracked (i.e., *r*_*j*_ ≥ AP), crow *i* approaches the hiding place (**pb**_*j*_^*t*^) of crow *j*; while if crow *j* knows that crow *i* is chasing it, crow *j* fools crow  *i* by going to a random location in the search space to protect its hiding place from detection. The mathematical expression is

(3) $$\begin{array}{c} \left\{\begin{array}{cc} x_{i}^{t+1}=x_{i}^{t}+r_{3}\cdot fl\cdot \left(pb_{j}^{t}-x_{i}^{t}\right), & r_{j}\geq \text{AP,}\\ \text{a random position,} & \text{else,} \end{array}\right. \end{array}$$

where *r*_*j*_ and *r*_3_ are random numbers with uniform distribution between 0 and 1, *fl* is the length of a crow's flight, AP is the perceptual probability of crow *j*, and **pb**_*j*_^*t*^ is the location where the current crow *j* stores food, which is equivalent to the historical optimal solution the current crow *j* has found.

CSA is a population-based optimization algorithm that is fairly simple with only two adjustable parameters (flight length *fl* and perceived probability AP), which in turn makes it very attractive for different engineering applications. In CSA, the parameters of perceived probability are directly used to control the diversity of the algorithm. Compared with genetic algorithm (GA), PSO, and harmony search algorithm (HS), CSA has fewer parameters to adjust and is easier to implement. Moreover, the individual in CSA has the possibility of reaching a total random position, and thus, it has stronger ability of exploring newly unknown regions. However, CSA has lack of the criterion for choosing the destination and the selection is done randomly between crows; besides, the flight length is a constant value. Such characteristics of CSA lead to a weaker ability of exploitation of current information compared with PSO, resulting in low search precision, high possibility of getting into the local optimum, and premature convergence, especially for multidimensional optimization problems.

### 2.3. Proposed CSO

To take advantage of both the PSO in exploitation and CSA in exploration, this study proposes a new crow swarm optimization algorithm (CSO). The movement of particles in CSO is influenced by the best position found so far by the particle itself and the best position identified so far by the whole swarm, as in PSO. Meanwhile, the particle keeps an eye on each other, i.e., there exists a possibility that its movement is determined by the best position identified by the whole swarm and the best solution found so far by another particle; in CSA, it tracks the position where another crow hides the food. The formula updating the flying velocity of crows in CSO is

(4) $$\begin{array}{c} \left\{\begin{array}{cc} v_{i}^{t+1}=\omega v_{i}^{t}+{\text{c}}_{3}r_{3}\left(pb_{j}^{t}-x_{i}^{t}\right)+{\text{c}}_{2}r_{2}\left(gb^{t}-x_{i}^{t}\right), & r_{j}\geq \text{AP,}\\ v_{i}^{t+1}=\omega v_{i}^{t}+{\text{c}}_{1}r_{1}\left(pb_{i}^{t}-x_{i}^{t}\right)+{\text{c}}_{2}r_{2}\left(gb^{t}-x_{i}^{t}\right), & \text{else,} \end{array}\right. \end{array}$$

where *c*_3_ represents the degree of the influence of individual *j* on individual *i* and *r*_3_ is a random number within [0, 1]. The updating velocity in equation (4) is also limited by **v**_min_ and **v**_max_.

When *r*_*j*_ ≥ AP, the individual *i* decided to track individual *j*, and the velocity of individual *i* is affected by inertia velocity, global optimal solution, and current optimal solution of individual *j*. Otherwise, the velocity of individual  *i* is composed of inertia velocity, global optimal solution, and local optimal solution of individual *i*. When the velocity of individual *i* is obtained, the position of individual *i* in the next iteration is calculated by equation (2).

**Figure 1:**
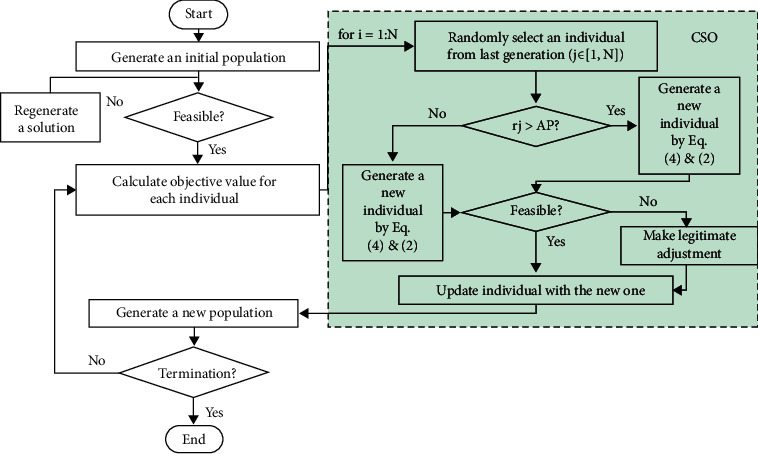
Flowchart of the proposed CSO.

Figure 1 shows the flowchart of the proposed CSO. It can be seen from Figure 1 that the difference between proposed CSO with other two algorithms mainly comes from the method of updating the particle speed and position. PSO pays more attention to the optimization efficiency and aims to be closer to the current optimal solution in the iterative process, thus resulting in a strong ability of exploiting the current known information, while CSA gives greater freedom to the algorithm to ensure the diversity of solutions, which produces greater ability of exploring unknown regions. The proposed CSO combines the advantages of both the PSO and the CSA to reach a better balance between increasing randomness and improving efficiency, i.e., between exploration and exploitation.

### 2.4. Individual Movement

**Figure 2:**
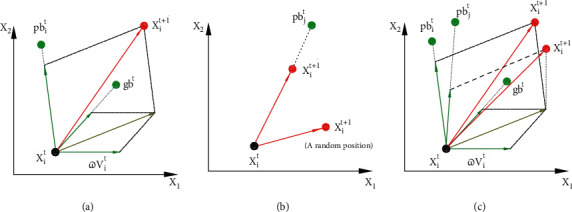
Schematics of how an individual updates its position in (a) PSO, (b) CSA, and (c) CSO, where the green spot indicates the current best position of an individual, the pink spot indicates the best position identified by the whole swarm, and the red spot indicates the position of the individual in the next iteration; the red arrows indicate the movement of the individual.

Individual movement directly affects the performance of the swarm intelligence algorithm.  Figure 2 shows the schematics of how an individual updates its position in (a) PSO, (b) CSA, and (c) CSO. The movement of the individuals in PSO is rather fixed and determined by its inertia, its current best position, and the best position identified by the whole swarm. Both CSA and CSO provide alternatives with a certain probability to better maintain the diversity of solutions. However, it can be seen that CSA is less efficient compared to CSO due to the divergence of the way the solutions update. CSO preserves the optimization efficiency of PSO with a possibility of exploring larger regions. In CSO, the larger the value of the probability parameter AP is, the stronger directional characteristic it has. When AP = 1, CSO degenerates into standard PSO; while when AP = 0, the individuals in CSO always randomly select the historical optimal solution of an individual in the population to track with less exploitation of other known information. It should be illustrated that when AP is equal to 0, CSO does not degenerate into CSA, but it retains the characteristics of rich diversity of CSA.

## 3. Experiments and Discussion

### 3.1. Standard Benchmark Functions

Extensive experiments are conducted on a set of well-known benchmark functions to ascertain the performance of the proposed CSO, including the global optimization problems shown in Table 1. The experimental settings are as follows: the maximum iteration number is 1000, and the swarm size is set to be 100; the parameters in equations (1) to (4) are set to be *c*_1_ = *c*_2_ = *c*_3_ = 2, *ω*=0.5, AP = 0.2, *fl* = 2, and **v**_max_ = [2]^D^.

In Table 1, *D* indicates the dimension of the problem and *x*_*i*_ represents the value of the decision vector in dimension *i*.

These test functions have included both unimodal functions (such as Sphere, Schwefel's problem 1.2, Step, and Rosenbrock) and multimodal functions (such as Ackely and Griewank). Unimodal functions with one global optimum and no local optima are used to investigate the exploitation level and the convergence rate of the algorithms, while multimodal benchmark functions with multiple local optima are used to test the ability of algorithms to avoid entrapment in local optima and explore new unknown regions.

In addition, a parameter representing the improvement efficiency is introduced to evaluate the progress of CSO compared with standard PSO or CSA:

(5) $$\begin{array}{c} \;\eta =\frac{\left({\text{optimal}}_{\text{PSO or CSA}}-{\text{optimal}}_{\text{CSO}}\right)}{{\text{optimal}}_{\text{CSO}}}. \end{array}$$

### 3.2. Optimization Efficiency

Table 2 shows the optimization results of the three algorithms for different target functions, respectively. In addition to the above-described three methods, recently, well-accepted bio-inspired optimization algorithms MBO and MS are also examined to better illustrate the performance of CSO. Each test conducts 25 trials to eliminate the influence of the initial population and enhance the reliability of the optimization results. Ten out of thirteen test functions show that CSO finds the best optimum values among compared to other algorithms. However, the performance of CSO on Rastrigin's function, Michalewicz function, and Griewank function is inferior to that of MS or MBO. For Rosenbrock function and CEC 2011 problem of parameter estimation for frequency-modulated (FM) sound waves, CSO has its best performance extremely close to 0, which is never reached by neither MS nor MBO. However, how to maintain this potential in each run still needs further research. It can be seen from the data that, in most cases, CSO performs better than both PSO and CSA, with not only a smaller value of the optimum but also less standard deviation. The results indicate that CSO has stronger optimization ability with higher stability. For some benchmark problems, the minimum value obtained by CSO is even different in magnitude from both PSO and CSA, indicating that the optimization performance of CSO has been greatly improved. CSA performs better in specific problems where the feasible region is relatively small, such as CEC2011 problem2. However, when the feasible range is in the hundreds of orders of magnitude, CSO's performance can degrade dramatically. Although we tried to change the parameters of the algorithm, it was time-consuming and often difficult to obtain the desired results.

**Figure 3:**
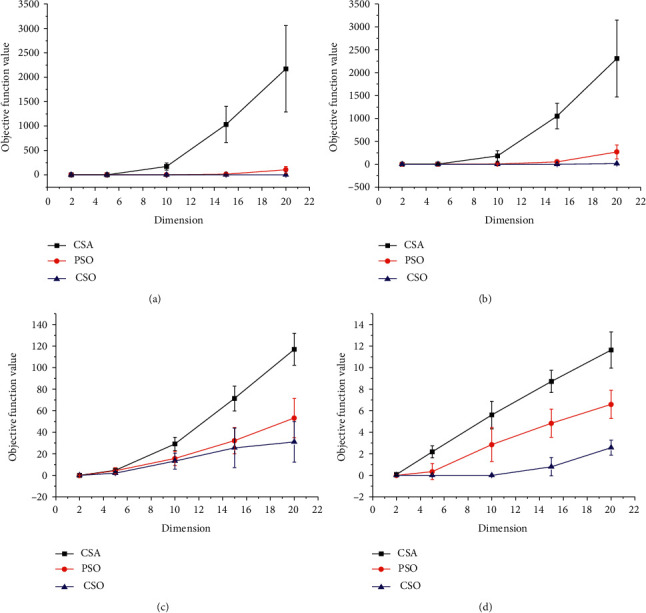
The performance of the three algorithms for (a) sphere function, (b) step function, (c) Rastrigin's function, and (d) Ackley function with different dimensions.

Figure 3 shows the variation of the optimums with the increase of the variables dimensions for (a) the unimodal problem of sphere function and (b) step function, as well as (c) the multimodal problem of Rastrigin's function and (d) Ackley function under other fixed conditions. It can be found from Figure 3 that CSO is able to maintain a relative high exploitation level for unimodal function with high dimensions. To improve the quality of the solution for high-dimensional problems, the size of the population is usually enlarged, which leads to a great increase of the calculation cost.  Figure 3(a) indicates that, for a 10D problem, the optimum resulting from CSO still remains in the order of 10^−23^, while those from CSA and PSO rise to 10^2^ and 10^−3^, respectively. Moreover, CSO also has the shortest error bars, which represent the stability of the algorithm. Figure 3 demonstrates that CSO performs superiorly for high-dimensional unimodal problems.

The improvement made by CSO on the multimodal problems varies with different problems. For Ackley function, Table 1 shows that the optimization efficiency of CSO is still significantly better than that of CSA and PSO. The three algorithms perform the worst when solving Rastrigin's problem, in which case, CSO is still the best among them. Table 1 also indicates that the improvement efficiency *η* of CSO is as high as 0.96 and 0.26 compared with CSA and PSO, respectively.  Figure 3(c) shows the optimization results of Rastrigin's function with different dimensions. It is clear that the optimization results degrade significantly as the dimension increases. Nevertheless, CSO has the weakest degradation among the three algorithms. The same trend could also be observed in Figure 3(d).

### 3.3. Global Search Abilities

**Figure 4:**
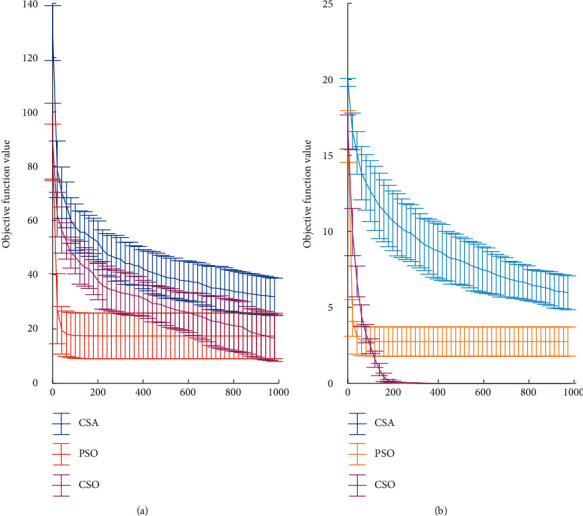
Optimization process for Rastrigin's function (a) and Ackley function (b) with a dimension of *D* = 10.

Figure 4 shows the evolution of the global optimum in the optimization process of the three algorithms. As suggested in Figure 4(a), PSO converges earliest in the iteration, and there is almost no improvement after 100 iterations. However, for CSA and CSO, improvements can still be observed even at the end of the iteration. Such phenomenon indicates that PSO is prone to premature convergence, while CSA and CSO still preserve the diversity of solutions to a certain extent during the optimization process, i.e., have a stronger ability of exploring new regions in the search space. It can be seen from Figure 4(a) that although CSA can avoid being trapped in local optima, and its ability to find a better solution is weak. In the later iteration, the solution obtained by CSO is smaller compared to CSA. To better investigate the optimization process, a concept of turning point is proposed here. When the optimum of a certain generation is better than that of the previous generation with a certain amplitude (in this study, it refers to the fourth decimal point or above of the objective value being improved), the current generation is defined as a turning point.  Table 3 shows the statistics of turning points for the three algorithms in 25 trials. For Rastrigin's function, the average number of the turning points of PSO is much higher than that of CSA and CSO, but most of them are centralized in the first 100 iterations and the change for each turn is small. As for CSA and CSO, the last turning point is over 800, verifying that both CSA and CSO have a better ability to jump out of the local optimal solution, i.e., the ability of exploration. Since CSA gives too much freedom to the algorithm, the amount of turning point is least, indicating its low efficiency. CSO possesses a moderate number of turn with relative high change, which makes it a more balanced algorithm.

Figure 4(b) shows the optimization process of the three algorithms for Ackely function. For PSO, there are several times when the optimum is trapped around 2. The optimization process of CSA looks like a staircase and improved every several iterations. It can be inferred that a certain number of iterations is still in need to get to 0. The best results are obtained from CSO, for which the optimums of all trails are quite close to 0. The calculated turning point in Table 3 shows that, after about 365 iterations, CSO almost reaches the global optimum value. The purple line (CSO) first sandwiched between the yellow line (PSO) and the blue line (CSA), and then, it passes these two lines coming to the bottom of the diagram in both Figures 4(a) and 4(b). CSA introduces an antitrack mechanism so that more randomness is given to the algorithm, which greatly reduces the possibility of entrapment in a local optimum at the expense of efficiency [13]. PSO employs local and global optimal solutions to direct the optimization process, so it has higher optimization efficiency but also higher probability of getting stuck. Based on the above results, it can be concluded that CSO absorbs the advantages of PSO and CSA while overcoming the shortcomings of both, so it is able to approach the global optimal solution quickly while maintaining a certain diversity of solutions.

### 3.4. Robustness to Parameter Settings

Table 4 is the optimization results of two unimodal functions, indicating the impact of the setting of parameter AP on the algorithm performance with other parameters unchanged. Given that AP ∈ [0, 1], when AP takes the values of the two endpoints, the optimization result is always the worst. For AP = 1, the result of CSO is close to that of PSO. For unimodal problems such as sphere functions, the performance of CSA deteriorates with the increase of AP, while for Schwefel's problem 1.2, the results of CSA is unstable and do not have obvious rule; thus, the tunning of AP is a big challenge.

**Figure 5:**
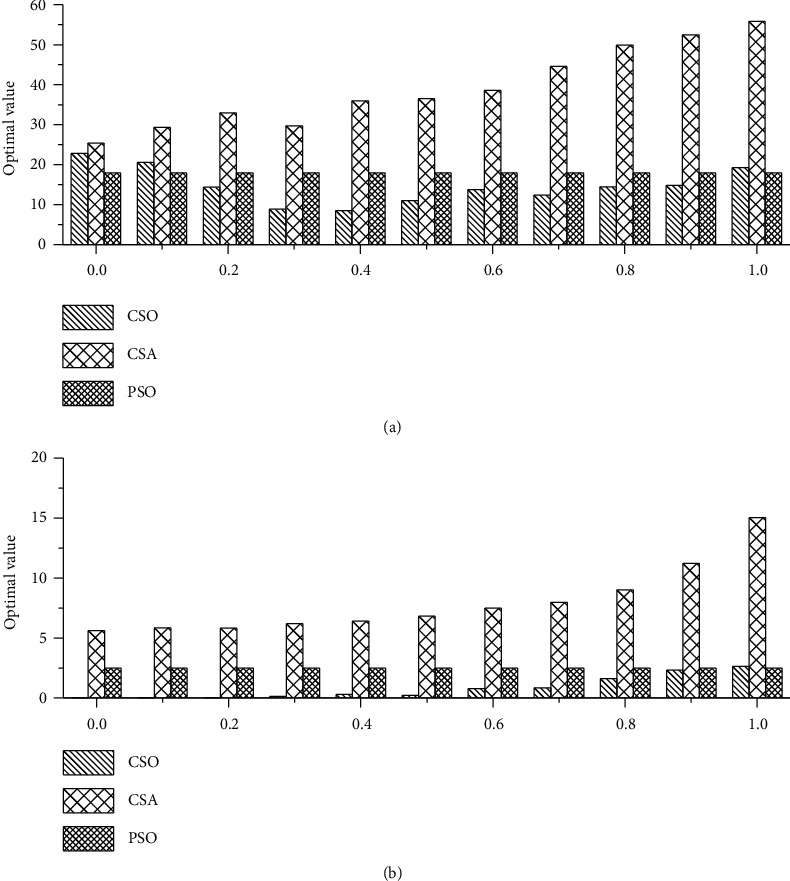
The impact of AP on solving multipeak problems: (a) Rastrigin's function and (b) Ackley function.

Figure 5 shows the impact of AP on solving more complex multimodal problems. It can be seen that the performance of CSO is not stable when AP is close to endpoints. For the case AP = 1, CSO degenerates into PSO. For CSA algorithm, when *fl* = 2, the optimal value of AP is concentrated between 0 and 0.2 [10]. If AP is too large, the optimization ability of CSA becomes quite weak. When the AP gets closer to 1, CSO gradually approaches PSO, and the algorithm can be easily trapped in a local optimum. Both Table 4 and Figure 5 indicate that the value of AP should be far from the two endpoints for CSO.

The impact of AP on the performance of CSO and CSA is discussed above given that *fl* = 2. *fl* is another important parameter for CSA algorithm. The combination of these two parameters together determines the optimization capability of CSA. Askarzadeh [10] has mentioned that the parameters of *fl* and AP played an essential role in the CSA search process as the solution obtained by the right parameter was far superior to the one using the improper value. For different optimization problems, it is necessary to choose among different parameters to get the reasonable optimal solution.

**Figure 6:**
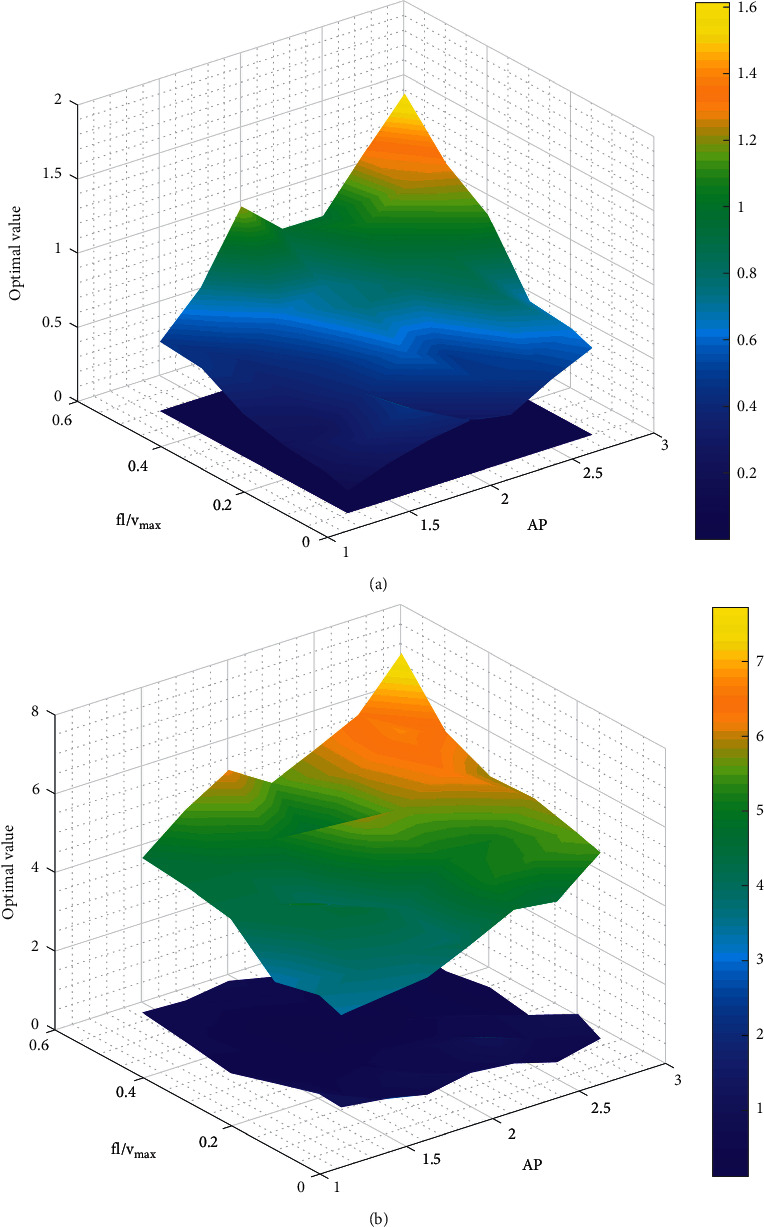
Optimization results of CSA and CSO with different parameter settings for (a) sphere function (*D* = 6) and (b) Rastrigin's function (*D* = 4) (the decision space is set to be [-10, 10]^D^).

Figure 6 compares the results obtained from CSA and CSO under different parameter combinations. In the previous discussion, it has been realized that a large AP would greatly undermine the optimization ability of CSA. Therefore, the upper bound of AP  is set to 0.5. Askarzadeh [10] also mentioned that the best choice of parameter *fl* may be around 2. Thus, the range of *fl* is set as [1.25, 2.75].  Figure 6(a) shows the optimization result of sphere function with dimension of 6, and Figure 6(b) is that of Rastrigin's function with dimension of 4. The upper surface of the figures represents the running result of CSA algorithm, while the lower surface is the optimized result of CSO. As parameters *fl* and **v**_max_ play the similar role in the optimization process as they both restrict the updating velocity of the solution, the parameter *v*_max_ (assuming **v**_max_ has the same value in each dimension, so *v*_max_ is treated as a scalar) in CSO is taken as *fl*. As can be explored from Figure 6, the upper surface is always steeper than the lower surface, indicating that the performance of CSA greatly depends on the setting of parameters. For the sphere function, of all parameter combinations applied in this study, the worst result (1.61) is 10 times worse than the best result (0.15). However, for CSO, all parameter combinations reach extremely close to 0, making almost a flat surface, i.e., CSO has stronger robustness to parameter settings compared with CSA. For multipeak Rastrigin's function, the standard deviations for CSA and CSO are 1.02 and 0.25, respectively, which also proves that CSO greatly reduces the dependency on parameters.

### 3.5. Application in Real-World Problem

Optimization algorithm is often used in the optimal operation of reservoir. This section describes the ability of the CSO algorithm to solve real-world problem. It is a short-term scheduling of a hydrothermal power system described in CEC 2011 competition. The optimal goal is to meet the load demand and reduce the cost in accordance with the various constraints put on the hydraulic systems and the power system networks. Hourly discharge management for four hydro units requires 96 variables, i.e., *D* = 96.

The objective function for this problem is to minimize the cost of thermal power plant. The operation is subjected to several limitations, including demand constraints, thermal generator constraints, hydro generator constraints, storage capacity constraints, and reservoir limit constraints. These constraints are added in the fitness function as penalty term. The detailed description and data could be found in [24].

**Figure 7:**
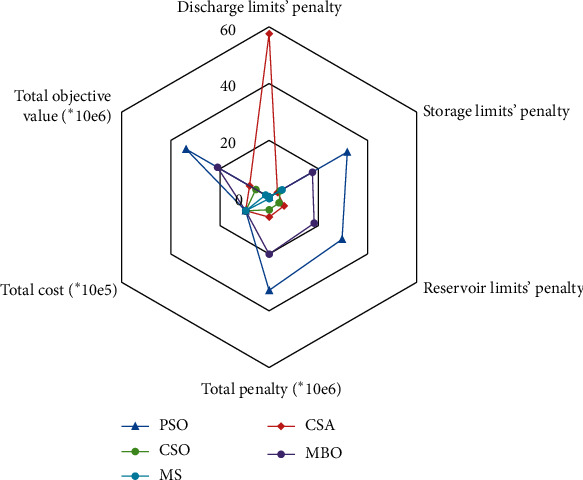
Optimization result of hydrothermal scheduling problem using different algorithms.

Each algorithm was run for 25 times, and the average value of the optimal solution for each run was calculated to get the results, as shown in Figure 7. From the figure, we can see PSO performs badly since several penalties being relatively large, leading to the worst total objective value. MBO occupies the second largest circle, indicating that its performance is slightly better than that of PSO. As for CSA, the total objective value is small compared to PSO, while it violates the requirements of the discharge to a large extent. The best result comes from MS, which takes up the smallest area. CSO also meets almost every requirement with slight penalty.  Table 5 shows the optimal and average values of 25 runs. The best total objective value comes from CSO and MS, which reduced by 57.6%, 70.9%, and 85.5% compared to CSA, PSO, and MBO, respectively. Optimized total cost does not make huge differences, and the penalty term is deterministic factor that influence the optimization results. Both CSO and MS could make every penalty equal or close to 0. Thus, it is believed that CSO has the same strength when tackling this problem, but sometimes it is trapped in local optima, which needs further improvements. As a conclusion, the operation scheme obtained by CSO could be a powerful alternative. Combined with above analysis, CSO may also be strong in real-world problems since it is explorative and efficient.

## 4. Conclusion

The balance between exploitation and exploration essentially determines the performance of a population-based optimization algorithm, which is also a big challenge in algorithm design. PSO is a popular bio-inspired optimization algorithm with strong ability in exploiting the existing information, i.e., every movement of the particles is under a certain guidance resulting from the previous knowledge, which leads to the weakening of the ability of exploring. CSA is a recent proposed algorithm with characteristics of simplicity, less parameters, and more randomness. The probability of flying totally randomly makes CSA has stronger ability of exploring new regions in the search space, while also results in a large computational cost and low search precision. This study proposes a new crow swarm optimization algorithm coupling PSO and CSA, which provides the individuals the possibility of exploring the unknown regions under the guidance of another random individual. The proposed CSO algorithm is tested on several benchmark functions, including both unimodal and multimodal problems with different variable dimensions. An example of its application in hydrothermal scheduling problem is also presented. The performance of the proposed CSO, the standard PSO, and CSA is evaluated by the optimization efficiency, the global search abilities, and the robustness to parameter settings. The results verify that the proposed CSO has better optimization ability compared with PSO and CSA, and it is less likely to fall into the local optimal solution than PSO algorithm and more efficient than CSA algorithm, i.e., CSO is good at approaching the global optimum quickly while maintaining a certain diversity of solutions during iteration. It can be concluded that the proposed PSO combines the advantages of PSO in exploitation and that of CSA in exploration. In addition, CSO has stronger robustness to the parameter *fl* and AP than CSA, which was regarded a major drawback of CSA previously. Although the proposed algorithm performs better than traditional PSO and CSO, it does sometimes become inferior to other algorithms. CSO, in this paper, is based on original PSO, but it could be applied in other modified version of PSO instead of traditional ones for possible further improvements. It is impossible for this algorithm to beat all the other algorithms, but it can be improved further, such as setting adaptive parameters, including subswarms. In addition to above algorithms, many other representative computational intelligence algorithms can be used to solve the problems, such as Harris hawks optimization (HHO) [25], which are not fully discussed in this paper. The source code of the proposed CSO algorithm is provided in this study, and more application and tests of CSO is welcome.

## Figures and Tables

**Table 1:** Formula of test functions.

Benchmark functions	Formula	Dimension	Up boundary	Lower boundary	Optima
Sphere	*f*(*x*_*i*_)=∑_*i*=1_^*D*^*x*_*i*_^2^	10	100	−100	0
Schwefel's problem 1.2	*f*(*x*_*i*_)=∑_*i*=1_^*D*^(∑_*j*=1_^*i*^*x*_*i*_)^2^	10	100	−100	0
Rosenbrock	*f*(*x*_*i*_)=∑_*i*=1_^*D*−1^[100(*x*_*i*+1_ − *x*_*i*_)^2^+(*x*_*i*_ − 1)^2^]	10	2	−2	0
Rastrigin's	*f*(*x*_*i*_)=∑_*i*=1_^*D*^[*x*_*i*_^2^ − 10 cos(2*πx*_*i*_)+10]	10	5.12	−5.12	0
Ackely	$f\left(x_{i}\right)=-20\cdot \;\exp\left(-0.2\sqrt{1/D\sum_{i=1}^{D}x_{i}^{2}}\right)-\exp\left(1/D\sum_{i=1}^{D}\text{cos}\left(2\pi x_{i}\right)\right)+20+e$	10	32	−32	0
Michalewicz	*f*(*x*)=−∑_*i*=1_^*D*^sin(*x*_*i*_)sin^20^(*ix*_*i*_^2^/*π*)	10	*π*	0	−9.66015
Branin	*f*(*x*)=(*x*_2_ − 5.1/4*π*^2^*x*_1_^2^+5/*πx*_1_ − 6)^2^				
10(1 − 1/8*π*)cos(*x*_1_)+10	2	15	−5	0.397887	
Griewank	$f\left(x\right)=\sum_{i=1}^{D}x_{i}^{2}/4000-\prod_{i=1}^{d}\cos\left(x_{i}/\sqrt{i}\right)+1$	10	600	−600	0
Normalised paraboloid	*f*(*x*)=*D*/4∑_*i*=1_^*D*^(*x*_*i*_ − 0.5)^2^	10	1	0	0
Step	*f*(*x*)=∑_*i*=1_^*D*^(*x*_*i*_+0.5)^2^	10	100	−100	0
Six hump camel bsck	*f*(*x*)=4*x*_1_^2^ − 2.1*x*_1_^4^+1/3*x*_1_^6^+*x*_1_*x*_2_ − 4*x*_2_^2^+4*x*_2_^4^	2	5	−5	−1.031628
CEC2011 problem1	CEC2011 problem1: parameter estimation for frequency-modulated (FM) sound waves [24]	6	6.35	−6.4	0
CEC2011 problem2	Lennard-Jones potential problem [24]	15	[4, 4, pi,…, $4+\frac{1}{4}\left\lfloor \frac{D-4}{3}\right\rfloor$]	[0, 0, 0,…, $-4-\frac{1}{4}\left\lfloor \frac{D-4}{3}\right\rfloor$]	

**Table 2:** Comparison of CSA, PSO, and CSO on test functions (25 trails).

Function	Algorithm	Optimum	Mean	Standard
*Sphere*	MBO	2.64*E* − 14	1.11*E* − 10	2.23*E* − 10
	MS	2.97*E* − 21	1.12*E* − 18	1.47*E* − 18
	CSA	3.21*E* + 01	1.63*E* + 02	8.38*E* + 01
	PSO	3.10*E* − 14	8.72*E* − 03	2.95*E* − 02
	**CSO**	**1.75 ** *E* − **24**	**4.27 ** *E* − **23**	**5.69 ** *E* − **23**

*Schwefel's*	MBO	1.62*E* − 08	2.40*E* + 03	2.75*E* + 03
	MS	5.45*E* − 20	1.91*E* − 18	2.66*E* − 18
	CSA	1.27*E* + 03	2.27*E* + 03	6.48*E* + 02
	PSO	4.55*E* − 05	9.96*E* − 01	2.10*E* + 00
	**CSO**	**2.46 ** *E* − **24**	**9.28 ** *E* − **23**	**1.22 ** *E* − **22**

*Rosenbrock*	MBO	1.63*E* − 02	1.64*E* + 00	1.81*E* + 00
	MS	3.28*E* + 00	**7.99 ** *E* − **01**	1.04*E* + 00
	CSA	3.09*E* − 01	8.27*E* − 01	**4.20 ** *E* − **01**
	PSO	1.98*E* − 05	6.31*E* + 00	1.39*E* + 01
	**CSO**	**2.50 ** *E* − **26**	1.79*E* + 01	2.77*E* + 01

*Ackley*	MBO	6.53*E* − 08	1.23*E* − 05	1.37*E* − 05
	MS	1.16*E* − 11	9.98*E* − 10	1.16*E* − 09
	CSA	3.50*E* + 00	5.82*E* + 00	1.29*E* + 00
	PSO	4.04*E* − 03	2.59*E* + 00	1.29*E* + 00
	**CSO**	**7.39 ** *E* − **13**	**2.90 ** *E* − **12**	**2.23 ** *E* − **12**

*Rastrigin's*	MBO	2.82*E* − 12	8.25*E* − 01	1.48*E* + 00
	MS	**0.00 ** *E* + **00**	**1.42 ** *E* − **16**	**4.82 ** *E* − **16**
	CSA	1.68*E* + 01	2.86*E* + 01	5.72*E* + 00
	PSO	4.08*E* + 00	1.84*E* + 01	9.29*E* + 00
	**CSO**	3.98*E* + 00	1.46*E* + 01	9.18*E* + 00

*Michalewicz*	MBO	−9.38*E*00	−**9.10 ***E* + **00**	**1.41 ** *E* − **01**
	MS	−9.58*E* + 00	−8.69*E* + 00	5.44*E* − 01
	CSA	−7.14*E* + 00	−6.31*E* + 00	4.16*E* − 01
	PSO	−9.12*E* + 00	−**7.74 ***E* + **00**	8.73*E* − 01
	**CSO**	−9.29*E* + 00	−7.64*E* + 00	9.12*E* − 01

*Branin*	MBO	**3.98 ** *E* − **01**	4.02*E* − 01	1.25*E* − 02
	MS	**3.98 ** *E* − **01**	**3.98 ** *E* − **01**	**1.11 ** *E* − **16**
	CSA	**3.98 ** *E* − **01**	**3.98 ** *E* − **01**	1.91*E* − 08
	PSO	**3.98 ** *E* − **01**	**3.98 ** *E* − **01**	**1.11 ** *E* − **16**
	**CSO**	**3.98 ** *E* − **01**	**3.98 ** *E* − **01**	**1.11 ** *E* − **16**

*Griewank*	MBO	6.66*E* − 15	2.61*E* + 00	5.22*E* + 00
	MS	**0.00 ** *E* + **00**	**0.00 ** *E* + **00**	**0.00 ** *E* + **00**
	CSA	1.37*E* + 00	2.52*E* + 00	7.30*E* − 01
	PSO	6.16*E* − 02	2.75*E* − 01	1.47*E* − 01
	**CSO**	2.96*E* − 02	1.29*E* − 01	1.03*E* − 01

Normalised paraboloid	MBO	3.23*E* − 15	4.28*E* − 11	7.50*E* − 11
	MS	1.39*E* − 14	9.55*E* − 14	6.63*E* − 14
	CSA	3.11*E* − 04	2.00*E* − 03	1.34*E* − 03
	PSO	1.27*E* − 18	3.00*E* − 08	1.21*E* − 07
	**CSO**	**4.92 ** *E* − **30**	**6.13 ** *E* − **28**	**1.98 ** *E* − **27**

*Step*	MBO	**0.00 ** *E* + **00**	**0.00 ** *E* + **00**	**0.00 ** *E* + **00**
	MS	**0.00 ** *E* + **00**	**0.00 ** *E* + **00**	**0.00 ** *E* + **00**
	CSA	1.80*E* + 01	1.72*E* + 02	1.08*E* + 02
	PSO	**0.00 ** *E* + **00**	3.56*E* + 00	2.98*E* + 00
	**CSO**	**0.00 ** *E* + **00**	**0.00 ** *E* + **00**	**0.00 ** *E* + **00**

*Six hump camel bsck*	MBO	1.17*E* − 16	1.70*E* − 13	4.04*E* − 13
	MS	5.13*E* − 29	4.00*E* − 24	8.99*E* − 24
	CSA	−**1.03 ***E* + **00**	−**1.03 ***E* + **00**	4.46*E* − 05
	PSO	−**1.03 ***E* + **00**	−**1.03 ***E* + **00**	**0.00 ** *E* + **00**
	**CSO**	−**1.03 ***E* + **00**	−**1.03 ***E* + **00**	2.09*E* − 07

*CEC2011 problem1*	MBO	1.18*E* + 01	1.95*E* + 01	3.55*E* + 00
	MS	1.67*E* − 02	1.91*E* + 01	6.20*E* + 00
	CSA	1.60*E* + 01	2.06*E* + 01	**2.53 ** *E* + **00**
	PSO	8.42*E* + 00	1.87*E* + 01	4.82*E* + 00
	**CSO**	**1.27 ** *E* − **21**	**1.14 ** *E* + **01**	8.68*E* + 00

*CEC2011 problem2*	MBO	−8.97*E* + 00	−7.85*E* + 00	8.96*E* − 01
	MS	−**9.10 ***E* + **00**	−**8.66 ***E* + **00**	7.03*E* − 01
	CSA	−8.92*E* + 00	−8.06*E* + 00	**4.57 ** *E* − **01**
	PSO	−**9.10 ***E* + **00**	−5.81*E* + 00	2.01*E* + 00
	**CSO**	−**9.10 ***E* + **00**	−6.24*E* + 00	2.17*E* + 00

*Note.* The bold fonts indicate the algorithm performs best for the corresponding problem.

**Table 3:** Statistics of turning points of 25 trials for test functions.

function	Parameter	Average number of turning points	Iteration time of the last turning point	Averaged optimal value	Averaged value change for each turn
*Rastrigin's*	PSO	86	112	19.07	0.84
	CSA	16	823	28.92	6.58
	CSO	36	839	11.72	3.08

*Ackley*	PSO	73	93	2.64	0.19
	CSA	32	922	6.18	0.46
	CSO	83	365	2.58e-12	0.20

**Table 4:** Algorithm performance with a different AP.

Sphere function	AP					
	0	0.2	0.4	0.6	0.8	1
CSO	1.29*E *−* *318	2.44*E *−* *323	2.85*E *−* *331	8.62*E *−* *342	5.22*E *−* *354	4.52*E *−* *3
CSA	122.51	202.91	318.31	408.18	881.13	3029.16

PSO	**1.42E ** * *−* *3**3**					

Schwefel's problem 1.2 function	AP					
	0	0.2	0.4	0.6	0.8	1

CSO	1.44*E *−* *318	3.91*E *−* *323	7.77*E *−* *331	1.94*E *−* *341	5.88*E *−* *354	8.28*E *−* *301
CSA	3452.62	2900.35	2986.59	2531.65	2660.23	3.23*E* + 03

PSO	**1.74**					

**Table 5:** Algorithm performance for hydrothermal scheduling problem.

Algorithm	Total objective value		Total cost		Total penalty	
	Minimum	Mean	Minimum	Mean	Minimum	Mean
CSA	2.22*E* + 06	7.96*E* + 06	9.42*E* + 05	9.53*E* + 05	1.28*E* + 06	7.00*E* + 06
PSO	3.23*E* + 06	3.79*E* + 07	9.46*E* + 05	9.58*E* + 05	2.26*E* + 06	3.28*E* + 07
CSO	9.41*E* + 05	5.41*E* + 06	9.41*E* + 05	9.57*E* + 05	18.9	4.46*E* + 06
MBO	6.48*E* + 06	2.10*E* + 07	9.43*E* + 05	9.58*E* + 05	5.52*E* + 06	2.01*E* + 07
MS	9.41*E* + 05	1.47*E* + 06	9.41*E* + 05	9.52*E* + 05	6.11	5.21*E* + 05

## Data Availability

The data used to support the findings of the study are available from the corresponding author upon request.
